# Key Factors Influencing Outpatient Satisfaction in Chronic Disease Care: Insights from the 2023 Korea HSES

**DOI:** 10.3390/healthcare13060655

**Published:** 2025-03-17

**Authors:** Yu-Jin Cha

**Affiliations:** Department of Occupational Therapy, Semyung University, Jecheon 27136, Republic of Korea; occujin@semyung.ac.kr

**Keywords:** chronic disease, experience, healthcare system, medical service, outpatient care, patient-centered care, patient satisfaction

## Abstract

**Background/Objectives**: The outpatient healthcare experiences of patients with chronic diseases significantly impact disease management and quality of life. Patient satisfaction with healthcare services serves as a critical indicator of the responsiveness of healthcare systems and the advancement of patient-centered care. This study aimed to identify key factors influencing patient satisfaction and propose strategies for improvement. **Methods**: Using secondary data from the 2023 Healthcare Service Experience Survey (HSES), we analyzed patient satisfaction and its associated factors among respondents aged 15 years and older from households nationwide. Through multiple regression analysis and statistical testing, we examined the impact of the healthcare facility type, sociodemographic characteristics, and healthcare service experiences on satisfaction levels. **Results**: Information provided by physicians and nurses, communication with healthcare providers, and shared decision-making processes had a significant impact on patient satisfaction. Satisfaction levels were highest in hospitals and lowest in clinics. Older patients and those who discontinued treatment due to the financial burden reported lower satisfaction. Notably, information provided by nurses had the most substantial positive influence on satisfaction. **Conclusions**: Delivering patient-centered outpatient healthcare services is essential in improving satisfaction and health outcomes. Enhancing the service quality, reducing financial burdens, and adopting digital platforms to promote patient engagement are critical measures. These approaches will strengthen chronic disease management systems and contribute to improving the overall quality of life of the population. The entire study focused on outpatient care.

## 1. Introduction

Improving the quality of healthcare services requires the positive transformation of the service experiences of patients. This entails not only enhancing the interactions between healthcare providers and patients but also making improvements to medical facilities and support services [1]. This entire study focuses on outpatient care. Tertiary hospitals are advanced medical institutions that provide highly specialized care, including complex surgeries and treatments for severe conditions. They serve as the highest-level referral centers, equipped with state-of-the-art medical technology and expert specialists. In contrast, hospitals are general medical facilities that offer a wide range of medical services, including inpatient and outpatient care, but do not provide the same level of specialized treatment as tertiary hospitals [2].

To realize the critical goal of strengthening health insurance coverage, it is essential to expand the financial resources of health insurance, supported by an increase in premiums. Achieving this requires building a public consensus, fostering a sense of solidarity among contributors as members of the community, and securing widespread public agreement on the policy [3]. The Organisation for Economic Co-Operation and Development (OECD) defines high-quality healthcare as being effective, safe, and responsive to patients’ needs while delivering patient-centered services [4]. Governments must understand how citizens, as both the beneficiaries of healthcare policies and contributors to health insurance funding, perceive healthcare systems and coverage expansion based on their sociodemographic and healthcare utilization characteristics. This understanding should serve as a foundation for the development of healthcare systems and health insurance policies that meet the practical expectations of the public [5].

The HSES, conducted annually by the Korea Institute for Health and Social Affairs (KIHSA) since 2017, evaluates citizens’ healthcare utilization experiences to assess the quality of healthcare services and produce internationally comparable patient experience indicators required by organizations such as the OECD [6]. The HSES has several distinguishing features. First, it captures qualitative aspects of public needs, such as patients’ experiences with healthcare services, satisfaction levels, and expectations regarding non-medical factors, which cannot be identified through conventional healthcare utilization statistics. Second, unlike traditional patient satisfaction surveys that focus only on the users of specific medical institutions, the HSES is a community-based comprehensive survey, offering greater objectivity and representativeness of the entire population [7].

The survey targets individuals aged 15 years or older from general households nationwide and includes self-report questionnaires on healthcare service experiences [8]. Many developed countries similarly conduct population-based representative surveys to understand patient experiences and improve the quality of healthcare services [9]. The HSES is shared with the OECD, facilitating cross-national and temporal comparisons, which necessitates the accurate understanding and interpretation of the reported data. Measuring patients’ experiences with healthcare services is crucial, as these experiences serve as key indicators of healthcare quality. However, as this is not an experimental study, achieving complete precision in measuring and comparing patient experiences remains inherently challenging [10]. Despite these limitations, the HSES is a valuable resource for the assessment of patients’ satisfaction and willingness to recommend healthcare facilities, particularly in the context of outpatient service experiences across both public and private hospitals.

Chronic diseases are long-term conditions that require continuous healthcare services. In South Korea, chronic diseases account for approximately 80% of the total deaths and constitute the majority of the leading causes of mortality, highlighting their significant impact [11]. Notably, a substantial proportion of individuals aged 65 and older suffer from two or more chronic conditions simultaneously, and the prevalence of such multimorbidity continues to rise alongside the aging population [12]. These conditions demand prolonged management and treatment, significantly affecting various aspects of patients’ lives. Therefore, studying outpatient healthcare service satisfaction among chronic disease patients is of critical importance [13]. Chronic disease patients often face limitations and stress in their daily lives, making their satisfaction with outpatient healthcare services highly significant. Understanding the factors influencing satisfaction among chronic disease patients can contribute to improving healthcare quality and fostering the delivery of patient-centered care [14].

Chronic disease patients with higher satisfaction levels regarding outpatient healthcare services are expected to demonstrate greater engagement in their treatment and management. Consequently, outpatient satisfaction is likely to have a significant impact on patients’ health outcomes and disease management. High levels of satisfaction among chronic disease patients can improve their quality of life, reduce their financial burdens, and enhance the efficiency and effectiveness of healthcare services. Therefore, understanding and improving outpatient satisfaction among chronic disease patients is essential [15].

Previous studies have primarily focused on factors influencing the awareness of specific healthcare services or the intention to utilize such services. However, there is a lack of research analyzing the factors affecting outpatient satisfaction among representative population groups [16]. As Korea’s healthcare environment is rapidly evolving due to the ongoing expansion of health insurance coverage, there is a pressing need to investigate public satisfaction with healthcare services using the latest data [6].

This study analyzes secondary data from the 2023 Korea Health Care Experience Survey to identify factors influencing outpatient satisfaction among chronic disease patients. The study aims to examine public perceptions of the healthcare system based on sociodemographic factors, chronic disease experiences, healthcare service utilization, and the characteristics of healthcare facility types. Additionally, it seeks to analyze key determinants of outpatient satisfaction. By doing so, this research underscores the importance of patient experiences as critical indicators of healthcare system responsiveness and the advancement of patient-centered care. It highlights the impact of positive service experiences on satisfaction, health outcomes, and overall patient engagement. The findings aim to provide actionable recommendations in terms of improving outpatient services and delivering patient-centered care while offering foundational insights for the development of healthcare policies that reflect public needs and expectations.

## 2. Materials and Methods

### 2.1. Study Setting

This study is a descriptive analysis utilizing secondary data from the 2023 HSES, a nationwide survey conducted by the Ministry of Health and Welfare (MHW) and the Korea Institute for Health and Social Affairs (KIHSA). The research examines patient participation experiences and satisfaction levels among chronic disease patients who utilized outpatient healthcare services. Additionally, it identifies key factors influencing satisfaction among these patients (Table 1).

### 2.2. Survey Method, Data Sources, and Study Population

The HSES is a nationwide annual survey targeting all household members aged 15 years and older from general households across the country. The survey retains a consistent structure, covering seven domains: health status, healthcare service utilization experiences, perceptions of the healthcare system, awareness of healthcare costs, health screening experiences, personal information, and household characteristics.

The 2023 HSES surveyed 14,910 individuals from 7000 households through in-person visits conducted from 24 July to 22 September 2023. The survey employs a computer-assisted personal interviewing (CAPI) method conducted by trained interviewers, supplemented by self-administered questionnaires when necessary. Sampling is based on enumeration districts from the Population and Housing Census, with strata defined by specific classification criteria. Households within the districts are selected using a probability-proportional-to-size systematic sampling method to ensure representativeness.

The original data for the 2023 HSES were obtained through the Microdata Integrated Service (MDIS) under Statistics Korea. The item name for the code type in the 2023 Healthcare Service Experience Survey is provided in Appendix A. Among the respondents, only individuals aged 15 years or older and covered by the National Health Insurance system were included in the analysis, excluding those under the Medical Aid program due to differences in healthcare and funding structures.

For this study, the target population included respondents from the HSES who reported outpatient visits to hospitals or clinics. Samples with missing responses for key variables, including non-responses, were excluded to finalize the study population. This study specifically examines five items related to outpatient service experiences and patient participation within the domain of healthcare service experiences.

### 2.3. Variables and Measures

Patient satisfaction was measured using the question, “Were you overall satisfied with the outpatient services you received?”. Responses were recorded on a 5-point Likert scale ranging from “very dissatisfied” to “very satisfied”. Based on prior research, satisfaction was categorized as “satisfied” for those who responded “very satisfied” or “somewhat satisfied”, while all other responses were classified as “dissatisfied” [17,18].

The chronic disease experience and healthcare utilization status were classified based on whether respondents had received treatment for chronic conditions (e.g., hypertension, diabetes, mental and behavioral disorders, respiratory diseases, heart disease, cerebrovascular diseases, neurological disorders, cancer, thyroid disorders, liver diseases, or chronic kidney disease) within the past year (“yes” or “no”). The number of chronic conditions was determined by the count of diseases treated within the past year, including major chronic diseases such as hypertension, diabetes, hyperlipidemia, joint disorders, tuberculosis, ischemic heart disease, cerebrovascular disease, and others.

Healthcare utilization was further categorized by outpatient service type: “hospitals”, “clinics”, “oriental medicine clinics”, “dental clinics”, “other”, and “non-utilization”. This study focuses on outpatient healthcare facilities that primarily provide care for chronic disease patients in South Korea. The analysis included three main facility types: tertiary hospitals, general hospitals, and clinics. These institutions are the primary providers of outpatient services for chronic disease management. To ensure a consistent and relevant dataset, certain facility types were excluded from the analysis. Specifically, oriental medicine clinics, dental clinics, and other types of healthcare facilities were not included, as they primarily offer specialized treatments that differ from conventional outpatient medical services for chronic conditions. Additionally, individuals classified under the “non-utilization” category were excluded, as this study focused on patient satisfaction among those who had utilized outpatient healthcare services. These exclusion criteria were applied to maintain the study’s focus on mainstream outpatient care settings and to ensure comparability across facility types.

Health screening experience was classified as either “yes” or “no”. Instances of forgoing medical care due to financial constraints were categorized as “yes”, “no”, or “unknown (not applicable)”. Patient participation experiences were evaluated using four dimensions adapted from the work of Lee et al. These included asking questions, seeking health information from a physician, seeking health information from a nurse, engaging in shared decision-making, and communicating with healthcare providers [19]. The dependent variable in this study, patient satisfaction, was defined based on outpatient service outcomes. Responses of “very satisfied” or “somewhat satisfied” were classified as “satisfied”, while all other responses were categorized as “dissatisfied”.

### 2.4. Data Analysis and Ethics

The data analysis for this study was conducted using SPSS 25.0 (IBM Corp., Armonk, NY, USA), with statistical significance set at *p* < 0.05. Variables and categories were based on prior research to identify factors influencing outpatient satisfaction among chronic disease patients. These variables were classified into patient characteristics and health-related factors [18,19,20,21].

Control variables included sociodemographic characteristics such as age, gender, education, employment, location, the medical insurance type, private health insurance, and the income quintile. Health-related factors included the perceived health status, the number of chronic diseases, experience of medical errors, health check-up experience, and instances of forgoing medical care due to the cost burden.

The analysis methods included descriptive statistics to examine the sociodemographic characteristics of participants using frequencies, percentages, and means. Second, differences in patient satisfaction based on patient participation experiences were analyzed using chi-squared (χ^2^) tests. While the chi-squared test is a widely used method for the analysis of categorical data, it is known to be sensitive to large sample sizes, often yielding statistically significant results even when the effect sizes are small. To address this limitation, we complemented our chi-squared test findings with multiple regression analysis and report effect sizes where applicable to ensure a more meaningful interpretation of the results. Third, multiple regression analysis was conducted to identify factors influencing outpatient satisfaction. To determine the independent variables with the greatest impact on outpatient satisfaction by healthcare facility type, standardized coefficients were analyzed, and variance inflation factors (VIF) were used to confirm the absence of multicollinearity issues.

This study utilized data from the 2023 HSES, which is publicly available through the Microdata Integrated Service (MDIS) website managed by Statistics Korea. The analyzed dataset contained no personally identifiable information. The study was approved for exemption from ethical review by the Institutional Review Board (IRB) of S University (IRB No. SMU-EX-2024-04-001).

## 3. Results

The demographic characteristics of the study population revealed that individuals aged 60 and older comprised the largest proportion at 42.2%, highlighting the significance of older adults as a key group in healthcare service utilization. Females accounted for 52.6% of the population, indicating higher representation compared to males. In terms of health-related characteristics, the enrolment rate in the National Health Insurance program was notably high at 90.7%, reflecting the program’s substantial role in improving healthcare accessibility. However, only 53.2% of respondents rated their subjective health status positively, while 46% reported having chronic diseases, indicating a considerable disparity between perceived health and actual health conditions (Table 2).

The analysis of the relationship between patient participation experiences and satisfaction in healthcare services revealed that 86.5% of respondents reported having participated in healthcare decisions, indicating that patient participation is a common practice. Among the methods of information delivery, receiving information through physicians was the most prevalent and served as a key factor influencing patient satisfaction. Notably, patients who actively engaged in communication and shared decision-making processes with healthcare providers demonstrated significantly higher satisfaction levels, a result that was statistically significant (*p* < 0.001). Conversely, limited patient participation was associated with lower satisfaction, supporting the finding that patients who actively asked questions reported higher satisfaction levels. Additionally, patients involved in the decision-making process exhibited a significant increase in satisfaction (*p* < 0.001), whereas those who did not participate showed relatively higher dissatisfaction rates with outpatient care (Table 3).

The analysis of differences in patient satisfaction across outpatient healthcare facility types revealed statistically significant variations among tertiary hospitals, hospitals, and clinics, with hospitals showing the highest satisfaction levels (*p* < 0.05). The post hoc analysis using Scheffé’s test indicated a statistically significant difference between hospitals and clinics, with clinics exhibiting relatively lower satisfaction. These findings suggest that the facility size and the quality of healthcare services play a critical role in shaping patient experiences (Table 4).

The analysis of the factors influencing outpatient satisfaction among chronic disease patients revealed varying effects of demographic characteristics, such as age, education level, and employment status, across models. Notably, age demonstrated a significant negative correlation with satisfaction (*p* < 0.05), with older patients reporting lower satisfaction levels. Among the methods of information delivery, receiving information through physicians and nurses had a positive impact on satisfaction, with information provided by nurses showing the highest explanatory power (β = 0.276, *p* < 0.001). Furthermore, participation in medical decision-making and effective communication with healthcare providers were key contributors to satisfaction, underscoring the importance of active patient engagement in improving the quality of healthcare services. Conversely, instances of forgoing medical care due to financial burdens negatively affected satisfaction. Additionally, the factors influencing satisfaction varied depending on the type of healthcare facility (Table 5).

## 4. Discussion

This study utilized data from the 2023 Korea Health Care Experience Survey to analyze factors influencing outpatient healthcare experiences and satisfaction among chronic disease patients. The primary objective was to examine the impact of sociodemographic factors and healthcare facility characteristics on patient satisfaction. The entire study focused on outpatient care.

The findings revealed that patient participation in healthcare services is common, highlighting the importance of active patient engagement in the medical environment. Among the methods of information delivery, receiving information through physicians had the most significant impact on satisfaction. Notably, patients who actively participated in communication and shared decision-making processes with healthcare providers reported higher levels of satisfaction, a result that was statistically significant.

Effective communication between healthcare providers and patients has been identified as a key factor in improving patient satisfaction, necessitating strategies for enhancement. First, healthcare professionals should be required to undergo patient-centered communication training and participate in regular workshops to strengthen their communication skills. Additionally, shared decision-making (SDM) should be expanded to encourage patient involvement in the treatment decision-making process, ensuring that their preferences are incorporated into clinical practice. Finally, extending outpatient consultation hours and improving the appointment scheduling system will help to guarantee sufficient consultation time between healthcare providers and patients, ultimately enhancing the overall quality of care.

Conversely, limited patient participation was associated with lower satisfaction, supporting the conclusion that asking questions and actively engaging with providers increases satisfaction. These results underscore the positive influence of patient involvement in medical decision-making on satisfaction and emphasize the need to further encourage patient participation to reduce dissatisfaction in outpatient care.

An analysis of the patient satisfaction differences by healthcare facility type revealed that satisfaction was highest in hospitals and relatively lower in clinics. The difference in satisfaction between hospitals and clinics was statistically significant, clearly demonstrating the disparity in satisfaction based on the facility type. The lower satisfaction in clinics is attributed to limited resources and differences in the quality of healthcare services.

There is a disparity in patient satisfaction between hospitals and clinics, with clinics showing relatively lower satisfaction levels, necessitating strategies for improvement. First, standardizing clinic services is essential to enhance healthcare quality, requiring the development and implementation of appropriate guidelines. Additionally, regular training and evaluation systems should be introduced for physicians and nurses in primary care facilities to strengthen the capabilities of these healthcare professionals. Lastly, implementing an online appointment system and optimizing the patient allocation processes will help to reduce waiting times, improve the efficiency of outpatient healthcare services, and ultimately enhance patient satisfaction.

Moreover, patient satisfaction factors vary across Asian regions due to differences in healthcare systems and socioeconomic environments. In East Asia (South Korea, Japan, Taiwan), the quality of medical services and the professionalism of healthcare providers were identified as key determinants of patient satisfaction, with clear communication and effective interactions playing a crucial role [22]. In Southeast Asia (Thailand, Malaysia, Indonesia), healthcare accessibility significantly influenced patient satisfaction, with urban–rural disparities in medical resources being a major contributing factor [23]. In South Asia (India, Bangladesh, Nepal), economic factors were the most significant, with high medical costs and limited insurance coverage lowering patient satisfaction [24].

In West Asia (Saudi Arabia, UAE), the modernization of medical facilities and personalized healthcare services were the primary drivers of patient satisfaction, particularly in private hospitals, where the service quality was notably high [25]. Overall, across Asian countries, the waiting times, interactions with healthcare providers, and service quality emerged as common determinants of satisfaction, although their relative impacts varied depending on the regional healthcare policies and economic conditions [22].

Similarly, variations in healthcare systems across different global regions influence patient satisfaction, particularly among chronic disease patients. In North America, the distinction between public and private insurance systems significantly affects access to care, with financial burdens being a major factor in patient dissatisfaction [26]. In Western European countries, the National Health Service (NHS) system in the United Kingdom provides universal healthcare services; however, long waiting times have been identified as a major source of dissatisfaction [27]. In contrast, Germany and France operate a mixed system of public and private health insurance, which ensures better healthcare accessibility and service quality, leading to higher levels of patient satisfaction [28].

East Asian countries, including South Korea, Japan, and Taiwan, emphasize healthcare quality and provider expertise, with strong national health insurance systems contributing to relatively high satisfaction levels. These regional differences underscore the importance of healthcare system design in shaping patient experiences and highlight the need for comparative studies to identify the best practices to improve chronic disease care satisfaction globally [22].

This study found that outpatient satisfaction was highest in hospitals and relatively lower in clinics, with the lower satisfaction in clinics attributed to limited resources and differences in service quality. However, previous studies have reported that small hospitals with fewer than 99 beds scored higher in terms of treatment processes and patient experiences compared to medium and large hospitals, which contrasts our findings. Given this discrepancy, further in-depth analysis is necessary. Further research is needed to compare and analyze the differences in patient satisfaction by hospital size (small hospitals, medium and large hospitals, and clinics) and to explore in depth the reasons that small hospitals received higher scores in previous studies.

As this study was based on a quantitative analysis using HSES data, it is crucial to complement it with qualitative research to gain a deeper understanding of patients’ actual experiences. Methods such as in-depth interviews and focus group discussions should be employed to collect detailed insights into patients’ personal experiences and their interactions with healthcare services. This approach will help to identify the underlying causes of both satisfaction and dissatisfaction. In particular, a more precise analysis is needed regarding the impact of interactions between healthcare providers and patients, methods of information delivery, and patient-centered decision-making processes. Exploring how patients build trust in healthcare services and identifying factors that contribute to lower satisfaction will provide valuable insights for the development of more effective improvement strategies.

To enhance the satisfaction in outpatient care settings, improvements in service quality and patient-centered approaches are essential. Previous research has indicated that small hospitals (fewer than 99 beds) tend to excel in treatment processes and patient experience scores compared to medium and large hospitals [29]. However, the findings of this study present a contrasting perspective, emphasizing the role of hospital size and service quality in shaping patient satisfaction. These results highlight the necessity for tailored healthcare service strategies that consider facility type and resource allocation.

Chronic disease patients can experience significant differences in disease management and quality of life depending on the quality of outpatient healthcare services. This study suggests that higher satisfaction with healthcare services is likely to enhance the efficiency of chronic disease management and underscores the need for improved accessibility and quality of healthcare services to achieve long-term health benefits [30]. These findings align with previous research on diabetes patients, which emphasized the importance of patient education and counseling in improving treatment satisfaction, ultimately contributing to better health outcomes and quality of life [31].

Patient participation extends beyond the provision of information, emphasizing the importance of active involvement in the treatment process to increase satisfaction. This aligns with findings that such engagement fosters a sense of security and strengthens patients’ commitment to the recovery process [32]. Active patient engagement is closely linked to improved satisfaction, highlighting the need to strengthen structured patient education and participation systems. First, regular educational programs should be implemented to enhance health literacy, covering topics such as chronic disease management, medication adherence, and lifestyle modifications. Additionally, a feedback system should be established to actively incorporate patients’ opinions and derive strategies to improve healthcare services. Lastly, digital healthcare platforms, including mobile apps and web-based services, should be introduced to allow patients to easily access and utilize their health information, thereby improving healthcare accessibility and usability.

This study demonstrates that information provided by physicians and nurses significantly enhances patient satisfaction, with information delivered by nurses showing the highest explanatory power [33,34]. Furthermore, patients who actively participated in effective communication and shared decision-making with healthcare providers reported higher satisfaction levels. These findings underscore the critical role of patient-centered care in delivering high-quality healthcare services.

This study found a negative correlation between age and satisfaction, indicating that older patients tend to report lower satisfaction levels [20,35]. This highlights the importance of developing tailored healthcare strategies for elderly patients, as patient satisfaction with healthcare services tends to decline with age, primarily due to communication barriers, limited accessibility, and difficulties in utilizing digital healthcare technologies.

To address this issue, it is essential to enhance tailored healthcare strategies for elderly patients. First, personalized counseling services should be provided, considering the physical and cognitive characteristics of older adults to better meet their individual healthcare needs. Additionally, healthcare providers should be trained to use age-friendly language and improve their communication skills to facilitate effective interactions with elderly patients. To overcome technological barriers, education on telemedicine and electronic health record (EHR) usage should be offered. Furthermore, hospital shuttle services should be expanded to improve accessibility for elderly patients, who face challenges in using public transportation.

Cases where financial burdens led to the forgoing of medical care were identified as significant factors reducing satisfaction. Moreover, the factors influencing satisfaction varied across healthcare facility types, emphasizing the need for differentiated approaches based on facility characteristics. Economic factors play a crucial role in patient satisfaction, and patients facing high medical expenses often forgo necessary healthcare services. To address this issue, policies should be implemented to expand health insurance coverage and support the continuous treatment of chronic disease patients. Additionally, financial counseling services should be provided to inform patients about the available financial assistance programs and medical expense reduction benefits, helping to alleviate their economic burdens. In particular, the activation of medical fee reduction programs for low-income patients is essential to bridge the gap in healthcare accessibility between high- and low-income groups and to create a more equitable healthcare environment.

These findings align with prior research showing that financial burdens from medical debt significantly decrease patient satisfaction and discourage individuals from seeking preventive or routine healthcare services [36]. Such results underscore the necessity of strengthening health insurance coverage and improving economic accessibility, requiring the development of sustainable policies. Patient-centered outpatient care is essential in enhancing satisfaction and health outcomes and can be successfully implemented through improved information delivery, education, and structured opportunities for active participation.

This study highlights the critical role of patient-centered outpatient care in improving patient satisfaction and health outcomes, emphasizing the importance of fostering patient participation and communication in healthcare policy development. Enabling patients to engage in goal setting requires the provision of information, education, and structured opportunities for involvement. These findings align with previous research showing that healthcare providers’ motivation and effective patient engagement strategies are essential in successfully implementing patient-centered care [37].

As this study utilized secondary data, which may impose certain limitations on variable selection and interpretation, future research should incorporate both quantitative and qualitative methods for deeper analysis. Specifically, comprehensive analyses and intervention studies reflecting diverse aspects of patient experiences are necessary to produce more detailed and actionable insights. The observation that limited patient participation correlates with lower satisfaction suggests the need to explore digital platforms to encourage patient engagement. Such efforts can strengthen chronic disease management systems and promote sustained healthcare utilization in an aging society. Furthermore, these measures will enhance equity in healthcare and contribute to a more positive healthcare experience for the population.

## 5. Conclusions

This study analyzed the factors influencing outpatient healthcare experiences and satisfaction among chronic disease patients using data from the 2023 Korea Healthcare Experience Survey. Communication and shared decision-making between patients and healthcare providers were identified as key factors significantly enhancing satisfaction. Satisfaction was highest in hospitals and comparatively lower in clinics, with the lower satisfaction in clinics attributed to limited resources and differences in service quality. Among sociodemographic factors, older patients reported relatively lower satisfaction levels, underscoring the need for targeted strategies for this population. Financial burdens were a major factor reducing patient satisfaction, emphasizing the necessity of strengthening health insurance coverage. Patient-centered care—characterized by improved information delivery, education, and opportunities for active participation—was confirmed as critical in enhancing satisfaction. Notably, information provided by nurses had the greatest positive impact on satisfaction. Addressing financial barriers through policy interventions is essential to mitigate dissatisfaction. The findings highlight the importance of adopting patient-centered approaches to improve satisfaction and establish a sustainable healthcare environment, particularly for chronic disease management and quality of life enhancement. This study underscores that patient-centered outpatient care is vital in improving both satisfaction and health outcomes and calls for a greater emphasis on patient participation and effective communication in healthcare policy development.

## Figures and Tables

**Table 1:** Research model.

Control Variables		Independent Variables		Dependent Variables
		Outpatient Care		
Patient characteristics Age Gender Education Employment Location Medical insurance Private health insurance Income quintile	Health-related characteristics Perceived health status Number of chronic diseases Experience of medical errors (side effects of drugs) Outpatient medical institution type Health check-up experience Cost burden abandonment of visit (treatment)	Experience of patient participation Asking questions Finding health information through a physician Finding health information through a nurse Engaging in shared decision-making Engaging in communication with healthcare providers	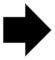	Satisfaction with outpatient care

**Table 2:** Patient and health-related characteristics of study participants.

Characteristics	Variable	Category	Satisfaction with Outpatient Care						χ^2^
			Satisfaction		Dissatisfaction		Total		
			N	(%)	N	(%)	N	(%)	
Patient characteristics	Age (yr)	15–19	167	87.9	23	12.1	190	(100)	<0.001 *
		20–39	1446	91.4	137	8.6	1583	(100)	
		40–59	3143	93.1	231	6.9	3374	(100)	
		≥60	3898	95.1	200	4.9	4098	(100)	
	Gender	M	3790	93.6	261	6.4	4051	(100)	0.862
		F	4864	93.6	330	6.4	5194	(100)	
	Education	Elementary school	634	95.8	28	4.2	662	(100)	<0.001 *
		Middle school	4622	94.4	276	5.6	4898	(100)	
		High school	3398	92.2	287	7.8	3685	(100)	
	Employment	Employee	3440	92.8	267	7.2	3707	(100)	<0.001 *
		Employer	1808	94.8	98	5.2	1906	(100)	
		Housewife	1945	93.6	133	6.4	2078	(100)	
		Student	358	88.8	45	11.2	403	(100)	
		Unemployed	919	95.9	39	4.1	958	(100)	
		Others	184	95.3	9	4.7	193	(100)	
	Location	Urban	6691	93.1	493	6.9	7184	(100)	<0.001 *
		Rural	1963	95.2	98	4.8	2061	(100)	
	Medical insurance	National Health Insurance	8381	93.6	572	6.4	8953	(100)	0.935
		Medical Aid	273	93.5	19	6.5	292	(100)	
	Private health insurance	Yes	6129	92.7	483	7.3	6612	(100)	<0.001 *
		No	2525	95.9	108	4.1	2633	(100)	
	Income quintile (grade)	Ⅰ	2101	94.2	129	5.8	2230	(100)	0.367
		Ⅱ	1731	93.7	124	6.3	1855	(100)	
		Ⅲ	1742	93.2	127	6.8	1869	(100)	
		Ⅳ	1518	94.2	93	5.8	1611	(100)	
		Ⅴ	1562	92.9	118	7.1	1680	(100)	
Health-related characteristics	Perceived health status	Good	4917	93.9	320	6.1	5237	(100)	0.316
		Fair	2785	93.0	208	7.0	2993	(100)	
		Bad	952	93.8	63	6.2	1015	(100)	
	Number of chronic diseases	0	4999	92.6	399	7.4	5398	(100)	<0.001 *
		1–2	3359	95.1	175	4.9	3534	(100)	
		≥3	296	94.6	17	5.4	313	(100)	
	Experience of medical errors (side effects of drugs)	Yes	6774	95.0	354	5.0	7128	(100)	<0.001 *
		No	694	87.4	100	12.6	794	(100)	
		Not aware (not applicable)	1186	89.7	137	10.3	1323	(100)	
	Health check-up experience	Yes	5094	93.9	331	6.1	5425	(100)	0.172
		No	3560	93.5	260	6.5	3820	(100)	
	Cost burden abandonment of visit (treatment)	Yes	189	93.1	14	6.9	203	(100)	0.001 *
		No	8406	93.3	565	6.7	8971	(100)	
		Not aware (not applicable)	59	83.1	12	16.9	71	(100)	

Note. Income quintiles were classified into five groups—the first, second, third, fourth, and fifth quintiles—based on the provided data. The first quintile represents the households with the lowest income, while the fifth quintile corresponds to those with the highest income. * indicates statistical significance based on the chi-squared test (* *p* < 0.05).

**Table 3:** Level of experience of patient participation and satisfaction with outpatient care (N = 8416).

Variable		Category	Satisfaction with Outpatient Care						χ^2^
			Satisfaction		Dissatisfaction		Total		
			N	(%)	N	(%)	N	(%)	
Experience of patient participation	Asking questions	Participated	7999	86.5%	417	4.5%	8416	(100)	<0.001 *
		Did not participate	655	7.1%	174	1.9%	829	(100)	
	Finding health information through a physician	Participated	8155	88.2%	427	4.6%	8582	(100)	<0.001 *
		Did not participate	499	5.4%	164	1.8%	663	(100)	
	Finding health information through a nurse	Participated	8138	88.0%	356	3.9%	8494	(100)	<0.001 *
		Did not participate	516	5.6%	235	2.5%	751	(100)	
	Engaging in shared decision-making	Participated	7952	86.0%	397	4.3%	8349	(100)	<0.001 *
		Did not participate	702	7.6%	194	2.1%	896	(100)	
	Engaging in communication with healthcare providers	Participated	7356	79.6%	319	3.5%	7675	(100)	<0.001 *
		Did not participate	1298	14.0%	272	2.9%	1570	(100)	
Types of medical institution	Tertiary hospital		514	5.6%	24	0.3%	538	(100)	0.037 *
	Hospital		1270	13.7%	104	1.1%	1374	(100)	
	Clinic		6870	74.3%	463	5.0%	7333	(100)	

* indicates statistical significance based on the chi-squared test (* *p* < 0.05).

**Table 4:** Differences in patient satisfaction based on types of outpatient care facilities.

Comparison	Mean Difference	Standard Error	*p*-Value	95% Confidence Interal	
				Lower	Upper
Tertiary hospital vs. hospital	0.031	0.012	0.044 *	0.001	0.062

* *p* < 0.05.

**Table 5:** Factors influencing outpatient satisfaction among patients with chronic diseases.

Variable	Model 1: Tertiary Hospital (N = 538)			Model 2: Hostipal (N = 1374)			Model 3: Clinic (N = 7333)			Model 4: Total (N = 9245)		
	β	t	VIF	β	t	VIF	β	t	VIF	β	t	VIF
Constant	0.290 *	2.318		−0.071	−0.770		−0.032	−0.766		−0.005	−0.119	
Age	−0.093 *	−1.975	1.344	−0.040	−1.339	1.437	−0.041 **	−3.212	1.434	−0.043 ***	−3.702	1.450
Education	−0.107 **	−2.510	1.086	0.020	0.782	1.054	0.011	1.036	1.082	0.009	0.943	1.070
Employment	−0.011	−0.256	1.250	0.017	0.620	1.309	0.021	1.812	1.175	0.019	1.835	1.206
Location	0.032	0.739	1.145	0.059 *	2.284	1.067	−0.020	−1.832	1.067	−0.002	−0.272	1.069
Private health insurance	0.038	0.811	1.340	−0.013	−0.469	1.371	−0.021	−1.788	1.237	−0.017	−1.559	1.263
Number of chronic diseases (ref: 0)	0.068	1.563	1.157	0.024	0.864	1.297	0.010	0.836	1.404	0.016	1.409	1.439
Experience of medical errors (side effects of drugs)	−0.026	−0.616	1.067	0.060 *	2.327	1.083	0.012	1.126	1.080	0.020 *	2.043	1.074
Cost burden abandonment of visit (treatment)	−0.053	−1.285	1.021	0.018	0.724	1.026	0.031	2.893	1.015	0.024 *	2.471	1.015
Asking questions	−0.009	−0.212	1.309	0.011	0.377	1.495	0.059 **	4.917	1.233	0.049 ***	4.570	1.256
Finding health information through a physician	−0.022	−0.507	1.212	0.130 ***	4.553	1.299	0.091 ***	7.581	1.238	0.091 ***	8.470	1.236
Finding health information through a nurse	0.276 ***	6.263	1.161	0.217 ***	7.903	1.208	0.215 ***	17.627	1.260	0.219 ***	20.214	1.243
Engaging in shared decision-making	0.011	0.251	1.215	0.096 **	3.198	1.439	0.039 **	3.124	1.367	0.047 ***	4.173	1.358
Engaging in communication with healthcare providers	0.104 *	2.272	1.269	0.044	1.566	1.262	0.085 ***	6.947	1.270	0.078 ***	7.168	1.264
R^2^	0.120			0.147			0.130			0.127		
Adjusted R^2^	0.098			0.139			0.128			0.126		
F	5.485 ***			18.027 ***			83.829 ***			95.945 ***		

Values are presented as odds ratios (95% confidence intervals). * *p* < 0.05. ** *p* < 0.01, *** *p* < 0.001.

## Data Availability

Publicly available datasets have been used for this study. The data used to support the findings of this study were provided by the Korean National Statistical Office (KNSO) under license: Statistics Korea (https://kosis.kr) (accessed on 20 November 2024).

## Appendix A. 2023 Healthcare Service Experience Survey Summary

No.	Code Type Item Name
1	Household Serial Number
2	Eastern/Rural Area
3	Subjective Health
4	Chronic Disease (None)
5	Chronic Disease 1 (Hypertension)
6	Chronic Disease 2 (Diabetes)
7	Chronic Disease 3 (Mental and Behavioral Disorders)
8	Chronic Disease 4 (Respiratory Disease)
9	Chronic Disease 5 (Heart Disease)
10	Chronic Disease 6 (Cerebrovascular Disease)
11	Chronic Disease 7 (Neurological Disease)
12	Chronic Disease 8 (Cancer)
13	Chronic Disease 9 (Thyroid Disorders)
14	Chronic Disease 10 (Liver Disease)
15	Chronic Disease 11 (Chronic Kidney Failure)
16	Chronic Disease 12 (Others)
17	Healthcare Service Experience (Outpatient)
18	Healthcare Service Experience (Inpatient)
19	Healthcare Service Experience (None)
20	Outpatient Visit Reason
21	Outpatient Medical Institution Visit Year
22	Outpatient Medical Institution Visit Month
23	Outpatient_Visited Medical Institution Type
24	Outpatient_Regular Usage Status
25	Outpatient_Reason for Choosing Medical Institution
26	Outpatient_Doctor_Courtesy
27	Outpatient_Doctor_Easy to Understand Explanation
28	Outpatient_Doctor_Opportunity for Sufficient Questions
29	Outpatient_Doctor_Reflection of Patient Opinion
30	Outpatient_Doctor_Empathy for Anxiety
31	Outpatient_Doctor_Explanation of Precautions
32	Outpatient_Doctor_Sufficient Conversation
33	Outpatient_Doctor_Consultation Time
34	Outpatient_Nurse_Courtesy
35	Outpatient_Nurse_Easy to Understand Explanation
36	Outpatient_Medical Institution_Comfort
37	Outpatient_Medical Institution_Administrative Service
38	Outpatient_Medical Institution_Protection of Physical Exposure
39	Outpatient_Medical Institution_Protection of Personal Information
40	Outpatient_Medical Institution_Recommendation Intention
41	Outpatient_Safety_Identity Verification Before Consultation
42	Outpatient_Safety_Explanation of Injection Purpose
43	Outpatient_Safety_Hand Sanitization Before Injection by Medical Staff
44	Outpatient_Safety_Disinfection of Injection Site Before Injection
45	Outpatient_Safety_Explanation of Drug Side Effects
46	Outpatient_Safety_Concern About Infection
47	Outpatient_Safety_Ease of Checking Safety Facilities
48	Outpatient_Consultation Waiting Type
49	Outpatient_Consultation Waiting Days
50	Outpatient_Consultation Waiting Time (Hours)
51	Outpatient_Consultation Waiting Time (Minutes)
52	Outpatient_Treatment Outcome Satisfaction
53	Outpatient_Overall Satisfaction
54	Outpatient_Overall Satisfaction_Dissatisfaction Reason
55	Inpatient_Reason for Hospitalization (Diagnosis)
56	Inpatient_Hospital Admission Year
57	Inpatient_Hospital Admission Month
58	Inpatient_Hospital Stay Duration
59	Inpatient_Hospitalization for COVID-19 Treatment
60	Inpatient_Medical Institution Type
61	Inpatient_Reason for Choosing Medical Institution
62	Inpatient_Doctor_Courtesy
63	Inpatient_Doctor_Easy to Understand Explanation
64	Inpatient_Doctor_Opportunity for Sufficient Questions
65	Inpatient_Doctor_Reflection of Patient Opinion
66	Inpatient_Doctor_Response When Needed
67	Inpatient_Doctor_Empathy for Anxiety
68	Inpatient_Nurse_Courtesy
69	Inpatient_Nurse_Easy to Understand Explanation
70	Inpatient_Nurse_Response When Needed
71	Inpatient_Nurse_Discharge Explanation
72	Inpatient_Medical Institution_Comfort
73	Inpatient_Medical Institution_Administrative Service
74	Inpatient_Medical Institution_Hospital Life Guidance
75	Inpatient_Medical Institution_Protection of Physical Exposure
76	Inpatient_Medical Institution_Protection of Personal Information
77	Inpatient_Medical Institution_Night Noise in Hospital Room
78	Inpatient_Medical Institution_Recommendation Intention
79	Inpatient_Safety_Identity Verification Before Consultation
80	Inpatient_Safety_Explanation of Expected Hospital Stay Duration
81	Inpatient_Safety_Explanation of Injection Purpose
82	Inpatient_Safety_Hand Sanitization Before Injection by Medical Staff
83	Inpatient_Safety_Disinfection of Injection Site Before Injection
84	Inpatient_Safety_Explanation of Drug Side Effects
85	Inpatient_Safety_Concern About Infection
86	Inpatient_Safety_Fall Experience
87	Inpatient_Safety_Ease of Checking Safety Facilities
88	Inpatient_Service Usage Type
89	Inpatient_Hospitalization Waiting Days
90	Inpatient_Hospitalization Waiting Reason
91	Inpatient_Hospitalization Route
92	Inpatient_Pre-Hospitalization Experience with Another Institution
93	Inpatient_Pre-Hospitalization Institution Type_Hospital
94	Inpatient_Pre-Hospitalization Institution Type_Clinic
95	Inpatient_Pre-Hospitalization Institution Type_Oriental Medicine Hospital
96	Inpatient_Pre-Hospitalization Institution Type_Dental Clinic
97	Inpatient_Pre-Hospitalization Institution Type_Other
98	Inpatient_Pre-Hospitalization Number of Visits_Hospital
99	Inpatient_Pre-Hospitalization Number of Visits_Clinic
100	Inpatient_Pre-Hospitalization Number of Visits_Oriental Medicine Hospital
101	Inpatient_Pre-Hospitalization Number of Visits_Dental Clinic
102	Inpatient_Pre-Hospitalization Number of Visits_Other
103	Inpatient_Hospital Room
104	Inpatient_Discharge Room
105	Inpatient_Preference for Specific Room Upon Discharge
106	Inpatient_Grievance Handling Procedure Guide
107	Inpatient_Treatment Outcome Satisfaction
108	Inpatient_Overall Satisfaction
109	Inpatient_Caregiver Service Experience
110	Inpatient_Family Caregiver Experience
111	Inpatient_Family Caregiving Days
112	Inpatient_Caregiver Hiring Days
113	Inpatient_Average Daily Caregiving Cost
114	Inpatient_Overall Caregiving Service Satisfaction
115	Inpatient_Private Caregiver Dissatisfaction_Cost
116	Inpatient_Private Caregiver Dissatisfaction_Hiring
117	Inpatient_Private Caregiver Dissatisfaction_Service
118	Inpatient_Private Caregiver Dissatisfaction_Other
119	Healthcare System_Overall Awareness
120	Healthcare System_Trust
121	Healthcare System_Satisfaction
122	Healthcare System_Need for Improvement
123	Healthcare System_Need for Improvement_Willingness to Pay Additional Insurance Premiums
124	Cost Burden_Abandoning Visit (Treatment)
125	Cost Burden_Abandoning Treatment
126	Cost Burden_Abandoning Test
127	Cost Burden_Abandoning Prescription Medication
128	Cost Burden_Abandoning Purchase of Medication
129	Cost Burden_Experience of Bearing Medical Expenses
130	Cost Burden_Level of Paid Medical Expenses Burden
131	Overtreatment
132	Health Check-Up_Experience
133	Health Check-Up_Type_National
134	Health Check-Up_Type_Individual
135	Health Check-Up_Type_Corporate Support
136	Health Check-Up_Payment Status_National
137	Health Check-Up_Payment Amount_National
138	Health Check-Up_Payment Burden_National
139	Health Check-Up_Inconvenience Experience_National
140	Health Check-Up_Inconvenience_Waiting Time_National
141	Health Check-Up_Inconvenience_Uncleanliness_National
142	Health Check-Up_Inconvenience_Formal Examination_National
143	Health Check-Up_Inconvenience_Explanation_National
144	Health Check-Up_Inconvenience_Other_National
145	Health Check-Up_Overall Satisfaction_National
146	Health Check-Up_Payment Status_Individual
147	Health Check-Up_Payment Amount_Individual
148	Health Check-Up_Payment Burden_Individual
149	Health Check-Up_Inconvenience Experience_Individual
150	Health Check-Up_Inconvenience_Waiting Time_Individual
151	Health Check-Up_Inconvenience_Uncleanliness_Individual
152	Health Check-Up_Inconvenience_Formal Examination_Individual
153	Health Check-Up_Inconvenience_Explanation_Individual
154	Health Check-Up_Inconvenience_Other_Individual
155	Health Check-Up_Overall Satisfaction_Individual
156	Health Check-Up_Payment Status_Corporate
157	Health Check-Up_Payment Amount_Corporate
158	Health Check-Up_Payment Burden_Corporate
159	Health Check-Up_Inconvenience Experience_Corporate
160	Health Check-Up_Inconvenience_Waiting Time_Corporate
161	Health Check-Up_Inconvenience_Uncleanliness_Corporate
162	Health Check-Up_Inconvenience_Formal Examination_Corporate
163	Health Check-Up_Inconvenience_Explanation_Corporate
164	Health Check-Up_Inconvenience_Other_Corporate
165	Health Check-Up_Overall Satisfaction_Corporate
166	Gender
167	Age
168	Education Level
169	Activity Status
170	Type of Medical Coverage
171	Private Health Insurance Enrollment
172	Household Income (Quintiles)
173	Household Weighting Factor_Population
174	Household Weighting Factor_Sample
